# Genetic Variability in *CLU* and Its Association with Alzheimer's Disease

**DOI:** 10.1371/journal.pone.0009510

**Published:** 2010-03-03

**Authors:** Rita J. Guerreiro, John Beck, J. Raphael Gibbs, Isabel Santana, Martin N. Rossor, Jonathan M. Schott, Michael A. Nalls, Helena Ribeiro, Beatriz Santiago, Nick C. Fox, Catarina Oliveira, John Collinge, Simon Mead, Andrew Singleton, John Hardy

**Affiliations:** 1 Laboratory of Neurogenetics, National Institute on Aging, National Institutes of Health, Bethesda, Maryland, United States of America; 2 Center for Neuroscience and Cell Biology, University of Coimbra, Coimbra, Portugal; 3 MRC Prion Unit, Department of Neurodegenerative Disease, UCL Institute of Neurology, London, United Kingdom; 4 Reta Lila Weston Institute and Departments of Molecular Neuroscience and Neurodegenerative Disease, Institute of Neurology, London, United Kingdom; 5 Neurology Service, Coimbra University Hospital, Coimbra, Portugal; 6 Dementia Research Centre, Institute of Neurology, University College London, London, United Kingdom; Peninsula Medical School, United Kingdom

## Abstract

**Background:**

Recently, two large genome wide association studies in Alzheimer disease (AD) have identified variants in three different genes (*CLU*, *PICALM* and *CR1*) as being associated with the risk of developing AD. The strongest association was reported for an intronic single nucleotide polymorphism (SNP) in *CLU*.

**Methodology/Principal Findings:**

To further characterize this association we have sequenced the coding region of this gene in a total of 495 AD cases and 330 healthy controls. A total of twenty-four variants were found in both cases and controls. For the changes found in more than one individual, the genotypic frequencies were compared between cases and controls. Coding variants were found in both groups (including a nonsense mutation in a healthy subject), indicating that the pathogenicity of variants found in this gene must be carefully evaluated. We found no common coding variant associated with disease. In order to determine if common variants at the *CLU* locus effect expression of nearby (*cis*) mRNA transcripts, an expression quantitative loci (eQTL) analysis was performed. No significant eQTL associations were observed for the SNPs previously associated with AD.

**Conclusions/Significance:**

We conclude that common coding variability at this locus does not explain the association, and that there is no large effect of common genetic variability on expression in brain tissue. We surmise that the most likely mechanism underpinning the association is either small effects of genetic variability on resting gene expression, or effects on damage induced expression of the protein.

## Introduction

Alzheimer's disease (AD) is recognized as a complex and multifactorial disease, in which genetics represents a significant role. A small subset of early onset AD cases (EOAD) are associated with mutations in three genes (*APP*, *PSEN1* and *PSEN2*). Most cases present with late onset of the disease (LOAD) without familial aggregation and are said to be sporadic. Several studies have tried to uncover the genetic basis of these late onset cases, but until recently, the only well established genetic risk factor associated with LOAD was ApoE [1]. In the past five years, the development of platforms able to genotype millions of SNPs and of powerful analytical frameworks able to distinguish true associations, together with the completion of the International Human HapMap project, have revealed risk factors associated with a wide variety of complex disorders [2].

Recently, the two largest AD GWAS reported three genes (*CLU*, *PICALM and CR1*) reaching genome wide significance when studying over 16000 individuals. In these studies, the most significant hit (rs11136000) is located in an intron of *CLU*, on chromosome 8 [3], [4].

Clusterin or apolipoprotein J is a lipoprotein expressed in most mammalian tissues with higher levels in brain, ovary, testis and liver [5]. It has been suggested to be involved in a variety of physiological processes, such as, ongoing synapse turnover [6], apoptosis [7], [8], cytoprotection at fluid-tissue boundaries, membrane recycling during development and in response to injury and regulation of complement-mediated membrane attack complex [9], [10]. Accordingly, CLU interacts with several different molecules, including lipids, amyloid proteins, components of the complement membrane attack complex and immunoglobulins [9]. From a biological point of view, it seems to have a potential central role in the pathogenesis of Alzheimer's disease. When control brains are compared with AD brains, CLU mRNA is found to be significantly elevated in AD affected brain areas [10], [11]. Clusterin is one of the proteins found to be part of amyloid plaques, the main neuropathological hallmarks of AD [12], [13], [14], [15]. Clusterin specifically binds soluble Aβ in cerebrospinal fluid [16], [17] to form complexes able to cross the blood-brain barrier [18]. It has also been noted that reduced levels of ApoE and increased levels of CLU are correlated with the number of E4 alleles, suggesting a compensatory induction of CLU in the brain of AD individuals with the E4 allele of ApoE presenting low brain levels of ApoE [19].

Because of the genetic and biological evidence suggesting a role for CLU in AD, we sought to characterize the common genetic coding variability in this gene. To accomplish this, we compared the genetic variation found in exon 5 of *CLU* (where rs7982 is located), between a total of 849 AD cases and 1067 neurologically healthy controls. Additionally, since it is now clear that common risk alleles and rare mutations in the same gene may contribute to common sporadic and familial forms of the same disease, respectively; we searched for rare mutations that could be related to familial AD cases in our sample. For this reason, we sequenced the entire coding region of *CLU* in a total of 495 AD cases and 330 controls.

The absence of common coding variants able to explain the association with disease observed by Harold et al and Lambert et al, suggested a potential implication of differential gene expression or splicing changes of the gene [3], [4]. In order to determine if common variants within these regions effect expression of nearby (*cis*) mRNA transcripts we conducted an expression quantitative loci (eQTL) analysis for *CLU* genomic region, for gene expression in cortical tissues from a group of 174 neurologically normal controls (GEO Series GSE8919, Myers et al 2007).

## Results

### Sequencing Analysis

A total of twenty-four variants were found in both cohorts. From these, eleven were observed in only one subject (Table 1). These eleven variants include synonymous changes (c.126C>G, p.T42T; c.132G>A, p.A44A; c.279C>T, p.Y93Y; c.348C>T, p.N116N; c.438G>A, p.K146K; and c.879C>T, p.H293H), suggesting no functional change for the protein; non-synonymous variants observed in controls and AD patients (c.284A>G, p.N95S; c.764C>T, p.T255I and c.1013G>A, p.R338Q); a nonsense mutation identified in a control individual (c.40G>T, p.E14X); and an in-frame deletion (c.812_814delTCT, p.F272del) present in a control individual.

**Table 1:** Changes (synonymous and non-synonymous) found in *CLU* and observed in only one subject (AD case or control) in both series.

Variants seen in only one subject					Sample					
Location in the gene	DNA change	Protein change	Present in dbSNP	PolyPhen prediction of pathogenicity (PSIC score)	Diagnosis	Sex	Age	AAO	Fam hist	Origin
5′UTR	c.290G>A	N.A.	no	-	Ctrl	M	-	N.A.	N.A.	UK
1	c.40G>T	p.E14X	No	-	Ctrl	M	69	N.A.	N.A.	PT
1	c.126C>G	p.T42T	No	-	Ctrl	F	70	N.A.	N.A.	PT
2	c.132G>A	p.A44A	No	-	AD	M	76	73	Pos	PT
3	c.279C>T	p.Y93Y	rs9331898	-	MCI	F	78	-	-	PT
3	c.284A>G	p.N95S	No	Benign (1.298)	Ctrl	M	59	N.A.	N.A.	PT
3	c.348C>T	p.N116N	No	-	AD	F	59	59	Neg	PT
4	c.438G>A	p.K146K	No	-	Ctrl	F	69	N.A.	N.A.	PT
5	c.812_814delTCT	p.F272del	No	-	Ctrl	F	28	N.A.	N.A.	UK
5	c.879C>T	p.H293H	No	-	AD	F	73	72	-	PT
6	c.1013G>A	p.R338Q	No	Benign (1.471)	AD	F	51	-	-	PT

Nucleotide numbering reflects cDNA numbering with +1 corresponding to the A of the ATG translation initiation codon in the reference sequences (CLU: NM_001831.2). The initiation codon is codon 1. Genomic numbering refers to reference sequence NC_000008.9. Protein numbering refers to sequence NP_001822.2. AAO: age at onset; Fam hist: Family history; N.A.: not applicable; -: information not available. Prediction of pathogenicity was performed in silico using the PolyPhen software [27], [28], [29].

Ten variants were found in more than one individual in the Portuguese series and seven in the UK series. The genotypic frequencies for these variants were compared between cases and controls (Tables 2 and 3). From these, four variants were found in both cohorts (p.T255I, p.H315H, rs3216167 and p.D380D).

**Table 2 pone-0009510-t002:** Variants found in *CLU* observed in more than one individual in the Portuguese series.

Location in the gene	DNA change	Protein change	Present in dbSNP	Frequency affected	Frequency unaffected	P	OR	PolyPhen prediction of pathogenicity (PSIC score)
Exon 1	c.48C>A	p.S16R	No	0.01425	0.01878	0.5459	0.7517	Benign (1.125)
Exon 2	c.240C>T	p.D80D	rs9331892	0.005181	0.007653	0.6074	0.6736	-
Exon 5	c.945T>C	p.H315H	rs7982	0.3575	0.3716	0.6296	0.9429	-
IVS6	g.27517690 -/A	NA	rs3216167	0.2642	0.2269	0.1449	1.233	-
Exon 7	c.1105A>C	p.N369H	rs9331936	0.008174	0.002358	0.2469	3.507	Possibly damaging (1.557)
Exon 7	c.1110C>G	p.P370P	rs9331937	0.001362	0.002358	0.6975	0.5765	-
Exon 7	c.1138G>A	p.D380N	rs9331938	0.002725	0.002358	0.9059	1.156	Benign (0.322)
Exon 7	c.1140C>T	p.D380D	rs9331939	0.001362	0.004717	0.3092	0.2869	-
3′UTR	g.27511359 T/C	NA	rs3087554	0.1425	0.1302	0.5646	1.112	-
3′UTR	g.27511354 C/T	NA	no	0.007634	0.005208	0.5001	1.723	-

Nucleotide numbering reflects cDNA numbering with +1 corresponding to the A of the ATG translation initiation codon in the reference sequences (CLU: NM_001831.2). The initiation codon is codon 1. Genomic numbering refers to reference sequence NC_000008.9. Protein numbering refers to sequence NP_001822.2. Prediction of pathogenicity was performed in silico using the PolyPhen software [27], [28], [29].

**Table 3:** Variants found in *CLU* observed in more than one individual in the UK series.

Location in the gene	DNA change	Protein change	Present in dbSNP	Frequency affected	Frequency unaffected	P	OR	PolyPhen prediction of pathogenicity (PSIC score)
5′UTR	c.-229G>C	NA	no	0.34	0.27	0.11	1.50	-
Exon 5	c.701G>A	p.R234H	no	0.001	0.002	0.51	0.47	Possibly damaging (1.641)
Exon 5	c.764C>T	p.T255I	rs41276297	0.003	0.006	0.35	0.53	Benign (0.310)
Exon 5	c.945T>C	p.H315H	rs7982	0.38	0.40	0.41	0.93	-
Exon 5	c.965T>C	p.P322L	no	0.002	0.002	0.73	1.42	Possibly damaging (1.963)
IVS6	g.27517690 -/A	NA	rs3216167	0.34	0.25	0.04	1.68	-
Exon 7	c.1140C>T	p.D380D	rs9331939	0.01	0.02	0.68	0.68	-

Nucleotide numbering reflects cDNA numbering with +1 corresponding to the A of the ATG translation initiation codon in the reference sequences (CLU: NM_001831.2). The initiation codon is codon 1. Genomic numbering refers to reference sequence NC_000008.9. Protein numbering refers to sequence NP_001822.2. p values presented here are uncorrected for multiple testing(none is statistically significant after Bonferronni correction). Prediction of pathogenicity was performed in silico using the PolyPhen software [27], [28], [29].

No statistical significant differences were observed for any of the genetic variants found in more than one individual, between cases and controls.

In order to predict the impact in protein function of the non-synonymous variants found in *CLU*, we performed an *in silico* analysis using PolyPhen (Tables 1–3). From the eight coding, missense and non-synonymous changes found in *CLU*, three were considered to possibly affect the protein function (p.R234H, p.P322L and p.N369H) while the other five were predicted to be benign. Nonetheless, these three variants are most likely non-pathogenic, since they have been found both in cases and healthy controls (Tables 2 and 3).

**Figure 1:**
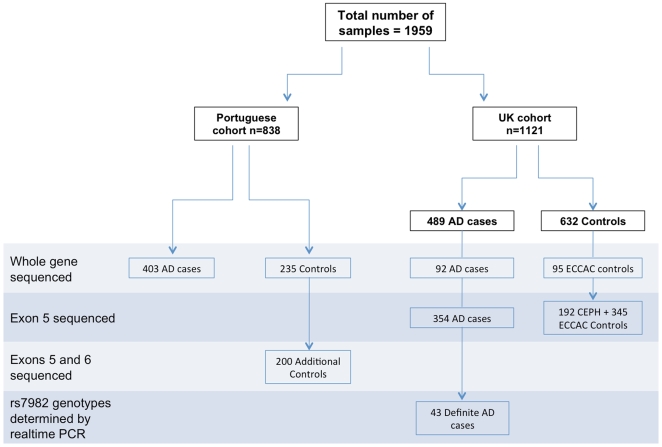
Summary of the samples used in this study and the type of genetic analysis of *CLU* performed in each subset. ECCAC - European Collection of Cell Cultures; CEPH - Centre d'Etude du Polymorphisme Humain.

Exons 5 (where the common H315H polymorphism is located) and 6 of *CLU* (where a non-synonymous change, p.R338Q, was found in a patient and was absent from controls) were screened in an additional set of 200 control individuals from Portugal. p.R338Q was not found in these additional 200 control samples (Figure 1).

### eQTL Analysis

**Figure 2:**
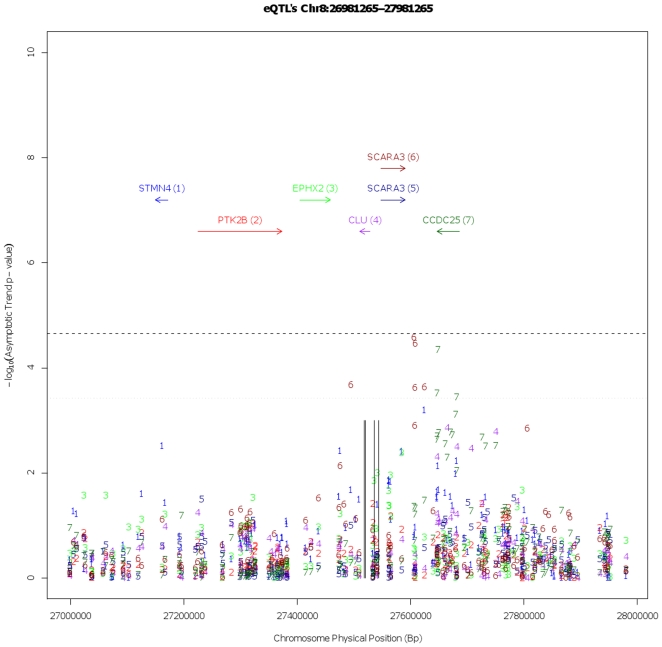
Manhattan plots for eQTL p-values +/− 250Kb of the previously AD associated regions near *CLU*. For this plot the x-axis represents the physical region of the chromosome and the y-axis is the –log_10_ of the asymptotic p-values from the eQTL analyses. The horizontal black dashed line represent the statistically significant threshold based on a Bonferonni correction for the number of SNPs and mRNA transcripts tested, while the black dotted represent the suggestive threshold based on the average number of SNPs tested per transcript. The relative positions of previously disease associated SNPs are denoted by vertical black lines. The individual p-values points for the SNP/Transcript tests are indicated by color and number so that the most significant values are the dark red ‘5’s for SCARA3 and the blue ‘6’s for CCDC25. In the plots the transcripts depicted are not all mRNA transcripts within the region but are instead the transcripts within the region with a probe present on the expression array and was well detected within the sample series.

eQTL analysis did not reveal any statistically significant results within the immediate region of AD associated loci for *CLU* (Figure 2). We were, however, able to confirm that *CLU* levels are higher in AD cases than in controls (GSE15222) [11], [20], though this is difficult to interpret because of the changing cellular composition of diseased tissue.

## Discussion

We sequenced the coding region *CLU* in 495 AD patients and 330 controls and exon 5 in a total of 849 AD cases and 1067 controls. This approach allowed us to find several new variants and, for some changes, compare the genotypic and allelic frequencies between cases and controls. Although variants in other exons are in LD with the previously associated SNPs, exon 5 was of particular significance, since this is the only exon where a potential functionally interesting coding SNP with a minor allele frequency (MAF) above 0.05 was identified (rs7982, p.H315H). Additionally, this was the only exon where coding variants were found in more than one individual in the UK series (p.R234H, p.T255I and p.P322L).

Our results are consistent with an association in the same direction for rs7982 (p.H315H) reported by Harold et al. The minor allele frequencies for this variant observed in the Portuguese and UK control series did not differ from those described by Harold et al (MAF in Portuguese controls: 0.37; MAF in UK controls: 0.40; MAF in Harold et al: 0.40). The failure to achieve statistical significance is most likely due to the small number of samples studied here, considering the marginal odds ratios associated with this variant (meta-analysis of the combined sample OR = 0.86, based on partially imputed genotypes in Harold et al) we only had ∼20% power to detect an effect of this magnitude in either cohort [3].

From the 14 variants found in more than one individual, none was found to be significantly associated with the development of AD. In the UK cohort, rs3216167 was found to have a nominal un-corrected p value of 0.04 (OR = 1.68, Table 3). A meta-analysis between the two cohorts revealed a similar value (p = 0.03 for random-effects meta-analysis, data not shown). This is a common SNP located in intron 6 that has previously been reported by Miwa Y. and colleagues to be associated with increased serum levels of total and LDL cholesterol, intima-media complex thickness of the common carotid artery and atherogenic plaque prevalence in Japanese hypertensive females [21]. Although a previous study of the distribution of polymorphisms in CLU in AD also found no individual coding or non-coding variants consistently associated with the disease, variants such as H369N and D380N have been reported to impact the levels of serum HDL cholesterol, HDL2, HDL3 and ApoA1 in African individuals [22], [23].

The sequencing of the gene allowed us to search for mutations that could be the cause of the disease in early onset AD cases. This is clearly of importance, since in many cases, common genetic variation associated with late onset common disease has been found in the same genes where mutations are known to cause early onset familial forms of the disease [24], [25]. A total of twenty-four variants was found, from which four were non-synonymous coding changes present in only one subject. Three of these changes (p.E14X, p.N95S and p.F272del) were found in controls and one (p.R338Q) was found in an early onset female AD patient. The screening of this variant in 200 additional Portuguese controls revealed a total of 1067 healthy individuals not harboring the p.R338Q change. Nonetheless, the lack of segregation data, the nonexistence of functional data for this variant and the *in silico* prediction by PolyPhen of non-pathogenicity, support a benign outcome for this genetic change in AD pathogenicity.

From the eight coding, non-synonymous changes found, three were predicted by PolyPhen to be possibly damaging (p.R234H, p.P322L and p.N369H). All of these were present in cases and controls, indicating that most probably these are also non-pathogenic variants. The finding of variants in controls, particularly the finding of a nonsense mutation in a 69 years old healthy individual, clearly adds to the requirement of an extended evaluation of the pathogenicity of each variant.

The sequencing approach used in this study, allowed us to compare not only one of the variants previously associated with AD (p.H315H) [3], but also, all the coding variants, the changes located in the exon-intron boundaries and in the 5′ and 3′ untranslated regions of *CLU*.

These largely negative results strongly suggest that: (1) the association signal reported by the two whole genome associations is unlikely to be caused by a common coding variant, such as is seen in APOE; (2) rare coding variants are not likely to be responsible for familial disease; and (3) that large eQTL effects in control tissue do not underlie the association. The possibilities that remain to explain the genetic association are that either there are small eQTL effecting expression (simulation studies suggest a study of this size could identify allelic effects of ∼20%) or that genetic variability in induced expression is the key issue.

## Materials and Methods

### Ethics Statement

All subjects included in this study, or their surrogates, gave written informed consent. This study and the use of these samples were approved for genetic research by: University College London Hospitals; National Hospital for Neurology and Neurosurgery; Coimbra University Hospital and National Institute of Health Ethics Committees. The study was conducted according to the principles of the Helsinki Declaration.

### Sequencing Analysis (Figure 1)

#### Portuguese series

After obtaining written informed consent from all participants or surrogates, blood samples were collected and DNA extracted from a total of 403 AD patients (62% of cases were women). All these were white with apparent Portuguese ancestry. The mean age of AD patients was 70 years old and mean age at onset was 67 years, ranging from 42 to 86 years. The genes associated with EOAD (*PSEN1*, *PSEN2* and *APP*) have been previously sequenced in all early onset cases (88 cases, with an age at onset before 65 years old) and no mutations were found [26]. Written informed consent was obtained from 235 neurologically normal and aged control subjects from Portugal (mean age at collection 68 years, 58% women). All controls were subjected to a neurological examination and found free of any symptoms suggestive of cognitive decline. This study and the use of these samples were approved for genetic research by the Coimbra University Hospital and National Institutes of Health Ethics Committees.

#### UK series

After obtaining written informed consent from all participants or surrogates, blood samples were collected from 489 AD patients (59% of cases were women). This study and the use of these samples were approved for genetic research by the University College London Hospitals and National Hospital for Neurology and Neurosurgery Research Ethics Committees. DNA was extracted using established protocols. A total of 476 cases were of UK origin, 5 were of other European origin, 2 were of non-European origin and 6 were of unknown origin. Patients were referred from the Dementia Research Centre, National Hospital for Neurology, Queen Square, UK, or other specialist cognitive teams in the UK, with either a clinical diagnosis of AD, or alternatively with cognitive impairment and referred for *APP*, *PSEN1* or *PSEN2* gene analysis. The mean age of onset was 54 years with a range of 21–78 years. One or more of the genes causally associated with EOAD (*PSEN1*, *PSEN2* and *APP*) and with prion disease (*PRNP*) were screened and found to be negative for mutations in 207 cases. *PRNP* alone was found to be negative for mutation in a further 88 cases, and the remaining 194 cases were not screened.

A total of 440 white healthy control DNAs were obtained from ECCAC (European Collection of Cell Cultures) and 192 unrelated individuals from the CEPH (Centre d'Etude du Polymorphisme Humain). Appropriate consent was available for all control individuals.

#### DNA sequencing and data analysis

Sequencing analysis of *CLU* (GeneID: 1191; isoform 1: NM_001831.2, which is the longest transcript of the gene, encoding 9 exons) was carried out using genomic DNA of a total of 638 Portuguese samples (403 AD cases and 235 neurologically normal controls) and 187 UK samples [92 AD patients and 95 UK healthy controls (ECCAC)]. All the coding exons plus the flanking intron-exon boundaries of *CLU* were PCR amplified using primers (available on request) designed using ExonPrimer software (http://ihg2.helmholtz-muenchen.de/ihg/ExonPrimer.html) and Roche FastStart PCR MasterMix polymerase (Roche Diagnostics Corp., IN). Each purified PCR product was sequenced using Applied Biosystems BigDye terminator v3.1 sequencing chemistry and run on an ABI3730xl (Applied Biosystems, CA) genetic analyzer as per manufacturer's instructions. The sequences were analyzed with Sequencher software, version 4.2 (Genecodes, VA).

Further 354 AD patients, 345 UK healthy controls (ECCAC) and 192 unrelated CEPH individuals were sequenced for exon 5 of *CLU* only. Genotypes for rs7982 were confirmed by realtime PCR using MGB allelic discrimination probes in all UK cases described above and an additional 43 definite AD cases.

In order to predict the impact in protein function of the non-synonymous variants found in *CLU* we performed an *in silico* analysis using PolyPhen [27], [28], [29].

SNPs were tested for association with Alzheimer's disease using logistic regression, assuming an additive model. These tests were performed using PLINK v1.07 [30]. In order to avoid any potential stratification effect, only the cases with known European ancestry were included in this analysis (13 samples from the UK series were excluded from the association analysis).

### eQTL Analysis

Since no gene expression data were available from the populations in which the sequencing analysis was performed, we used genotype and gene expression data from 174 neurologically normal controls from the United States, obtained from our previously published eQTL analysis [31]. Gene expression data are available at NCBI's Gene Expression Omnibus (GEO Series Accession, GSE8919) and genotype data are available from TGEN (http://www.tgen.org/research/neuro_gab2.cfm). Based on genotype data samples were checked for quality of genotyping based upon genotype call rate per sample, population outliers using Structure [32], [33] and Plink and for cryptic relatedness and gender discrepancies with Plink [30].

Genotype data were from the Affymetrix GeneChip Human Mapping 500 K Array Set, which assays 502,627 SNPs (Affymetrix, Santa Clara, CA). Genotypes were imputed based on HapMap data using the IMPUTE program resulting in genotype call for 1741175 SNPs after QC of imputed data. Based upon SNP selection criteria of Hardy-Weinberg Equilibrium p-value >0.001 and the presence of at least 3 minor homozygotes, 322 SNPs were selected for *cis* regional eQTL analysis near *CLU*, being the *cis* region defined as centered on the AD associated locus +/− 250 Kb. Gene expression data were from the Illumina HumanRef-8 v1 Expression BeadChip that assays 24,357 RefSeq mRNA transcripts (Illumina Inc., San Diego, CA). Expression data were rank invariant normalized [34], [35], [36] using Illumina's BeadStudio Gene Expression module. Expression mRNA transcripts were selected for analysis based upon whether they reliable detected within 95% of samples, are within the *cis* genomic regions of interest and where the probes designed to assay the mRNA transcript did not contain a common polymorphism that have been typed within the HapMap CEU population. This resulted in 6 mRNA transcripts being selected for analysis, including one CLU transcript. Prior to eQTL analysis each of the normalized transcript expression profiles was log_2_ transformed and adjusted for available covariates (age at death, gender, cortical region, post mortem interval, tissue bank source and RNA preparation/hybridization batch) by linear regression, the residuals of these regressions were then used as the quantitative trait for eQTL analysis.

The eQTL analysis was performed using Plink's [30] *assoc* function for quantitative traits, which correlates allele dosage with changes in the trait. To correct for the number of tests performed, a Bonferroni correction was applied to the asymptotic p-values based 322 SNPs tested against 6 transcripts in the *CLU* region. This Bonferroni correction for 1932 tests resulted in a significance threshold of 2.587992e-05 for asymptotic p-values.
